# Overview of Swine Congenital Malformations Associated with Abnormal Twinning

**DOI:** 10.3390/vetsci10090534

**Published:** 2023-08-23

**Authors:** Aris Pourlis, Georgios I. Papakonstantinou, Dimitrios Doukas, Vasileios G. Papatsiros

**Affiliations:** 1 Laboratory of Anatomy, Histology and Embryology, Faculty of Veterinary Medicine, School of Health Sciences, University of Thessaly, Trikalon 224, 43100 Karditsa, Greece; apourlis@vet.uth.gr; 2 Clinic of Medicine, Faculty of Veterinary Medicine, School of Health Sciences, University of Thessaly, Trikalon 224, 43100 Karditsa, Greece; vpapatsiros@vet.uth.gr; 3 Laboratory of Pathology, Faculty of Veterinary Medicine, School of Health Sciences, University of Thessaly, Trikalon 224, 43100 Karditsa, Greece; ddoukas@vet.uth.gr

**Keywords:** conjoined twins, teratology, pig, congenital malformations

## Abstract

**Simple Summary:**

This study investigated congenital malformations in pigs associated with anomalous twinning. Among the conjoined twins recorded, the most common defect was syncephalus thoracopagus or cephalothoracopagus. Some dicephalic and diprosopic anomalies were also recorded. Finally, some cases of thoraco-omphalopagus piglets were studied. The pathogenetic mechanisms of this disease, which is common in veterinary practice, are discussed. The significance of embryonic conjoined twins is frequently associated with dystocia.

**Abstract:**

A review of congenital malformations in swine relating to abnormal twinning was carried out. The aim was to describe and estimate these defects. Among the recorded twins, the most common defect was the *syncephalus thoracopagus* or *cephalothoracopagus*. A couple of dicephali and diprosopus congenital anomalies were also registered. At last, some cases of thoraco-omphalopagus piglets were surveyed. There was also a report of an acardiac twin (*hemiacardius acephalus*) and a case of a conjoined parasitic twin. The pathogenetic mechanisms of this condition, frequently reported in veterinary practice, are discussed. The importance of embryonic imperfect twinning is commonly associated with dystocia.

## 1. Introduction

Congenital malformations are observed in various cases of veterinary practice. The domestic pig is an animal model used to evaluate potential cause–effect relationships between the environment and congenital malformations. For this reason, swine have been used as a model for an in-depth study of human congenital anomalies [1]. In addition, pig production is widely propagated all over the world. The record of congenital defects is essential in veterinary medicine and pig husbandry. In farm animals, especially in swine, embryonic duplications represent one of the biggest groups of congenital anomalies and are a common cause of dystocia and the delivery of stillborn embryos [2]. Congenital duplications (conjoined twins, parasitic twins, or acardiac twins) are unique and interesting among congenital defects [2]. These duplications form a spectrum of structures which vary from slight duplication to near separation of two individuals. According to the degree, sites, and angle of fusion, they have wide external variation [1] and are classified as acardiac, conjoined symmetric, or asymmetric twins, and unequal conjoined twins (heteropagus or parasitic twins). However, in the *Nomina Embryologica Veterinaria* (2017) [3], a simpler classification of twinning was suggested. According to the latter, there are the Gemini acardiaci and the Gemini conjuncti. The Gemini conjuncti are further subdivided into Gemini conjuncti symmetrici and asymmetrici (Table 1).

## 2. Conjoined Twins

According to the *Nomina Embryologica Veterinaria* (NEV) the malformed conjoined twins are subdivided into two main categories, namely symmetric and asymmetric (International Committee on Veterinary Embryological Nomenclature 2017). Furthermore, both the symmetric and asymmetric twins are classified into three general conjunction groups: cranial, medial, and caudal conjunction. Classically, the conjoined twins can be divided into dorsal, lateral, and ventral conjunction types [4]. The caudal ventral conjunction comprises the ileoischiopagus, whereas the lateral conjunction is the *parapagus diprosopus* and *parapagus dicephalus*. The rostral ventral conjunction comprises the cephalopagus, thoracopagus, and omphalopagus. When the twins are joined dorsally, they are the craniopagus, rachipagus, and pygopagus. In more detail, the presentation of various genetic anomalies is provided and contrasted with the malformations identified in our case.

### 2.1. Diprosopus

Among the conjoined twins presented both by Wilder (1908) [5] and Bishop (1908) [6], two diprosopi twins were evaluated. The first, characterized as *diprosopus triophathalmos*, possessed two snouts, two normal external ears, two tongues, several teeth, and two laterally located eyes. The median eye was composed of a double eyeball. The second was characterized as *diprosopus tetrophthalmus*. A bilaterally symmetrical median eye composed of two eyeballs was observed. No median ear was present. Two snouts were separated.

In the case report of Thuringer (1919) [7], the diprosopus piglet possessed two snouts, two tongues which were fused at their base, two mandibles but a single nasopharynx, and a single epiglottis. In the face, four eyes were observed within three orbits. The head had two ears. Two separate cerebri, two cerebelli, and two fused medullae oblongatae, as well as a single spinal cord were noticed. The right and left lateral cerebral hemispheres and the corresponding lateral halves of the midbrains and pontes were normal in size. Fusion of the two cerebrospinal axes was affected in the lower part of the fourth ventricle and was limited to the medulla oblongata. In addition, complete bilateral duplication of the cranial nerves, from the first to the eighth pair, was observed. However, the entire skeleton (caudally to the first dorsal vertebra) and the heart were normal.

**Figure 1:**
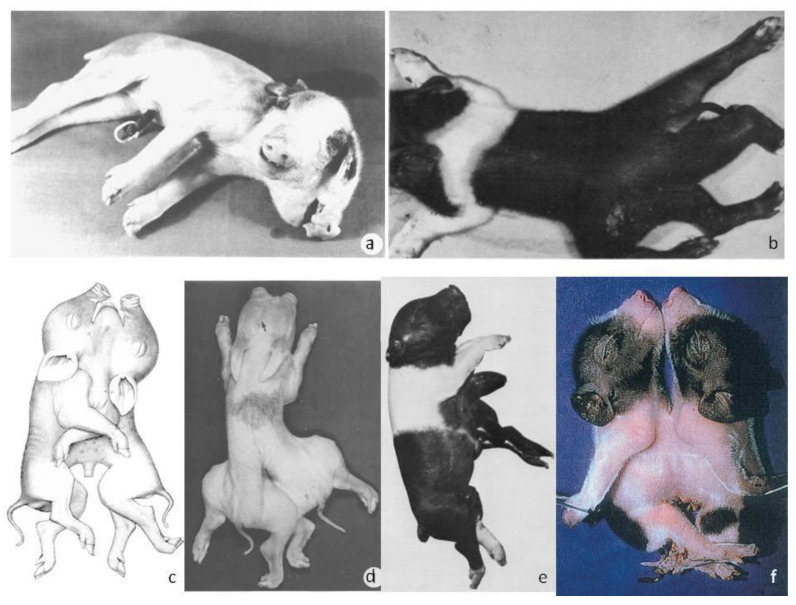
(**a**) Two-headed piglet [9], (**b**) Dipygus pig [10], (**c**) *Prosopothoracopagus disymmetros* [11], (**d**) *Diprosopus dipygus* [8], (**e**) *Epigastricus* parasite [10], (**f**) *Thoracopagus diplopagus* [12].

Later, diprosopus and dipygus porcine conjoined twins were studied [8]. In addition to the conjoining anomaly, these twins (Figure 1d) also exhibited ambiguous internal reproductive features. Two snouts, three eyes, and a single thorax were observed, while the twins were duplicated from the umbilicus caudally. Radiography revealed a single vertebral column in the cervical region. The vertebral columns were separated caudally from this point. There was a total of six limbs—one pair of forelimbs and two pairs of hindlimbs. Many medial structures were not developed in these twins, such as (a) medial cranial nerves V-XI1 that were absent or displaced although normal laterally, (b) medial palates that were present but shortened, and (c) medial mandibular rami that had folded back on themselves rostrally to form a midline mass between the two chins. Moreover, one lateral kidney and one lateral testis were noticed in each twin. Medial scrotal sacs were present but were devoid of a testis. Finally, a midline-like structure was observed that crossed between the twins and histological analysis revealed it to be dysplastic testicular tissue.

### 2.2. Dicephalus

**Figure 2:**
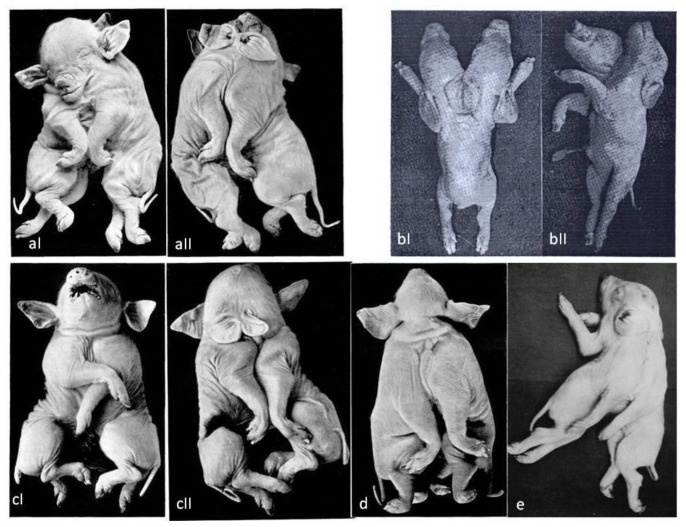
(**aI**,**aII**) *Cephalothoracopagus monosymmetros tetrophthalmus synotos tetrabrachius* [11], (**bI**,**bII**) *Dicephalus parapagus* [13], (**cI**,**cII**) *Cephalothoracopagus monosymmetros synotos tetrabrachius craniis a latere coalitis* [11], (**d**) *Cephalothoracopagus monosymmetros biauritus tetrabrachius* [11], (**e**) Syncephalus pig [10].

Groth (1964) [13] provided an illustration of a dicephalus piglet (Figure 2b) and Partlow et al. (1981) [9] provided a case report (Figure 1a). A normal body with two heads joined in the occipital region was noticed [10]. In addition, two complete snouts, four eyes, and three ears were noticed. The lower jaws were immobile because of overlapping mandibular rami. Although there was only one vertebral column, the bodies of the vertebrae, but not the neural arches, were doubled from the axis to T8. One thyroid gland and one larynx and hyoid apparatus were also observed. The two tongues were joined at their base just rostral to the single epiglottis. A completely split palate was observed in the right head but was only partially split in the left. The cranial nerves were normal and doubled except for IX, X, and XI. Moreover, fused brains were noticed at the pons–medulla junction. An anomalous midline tag of neural tissue resembling remnants of the medial halves of two nervous systems extended from this point to the level of T8.

### 2.3. Syncephalus Thoracopagus or Cephalothoracopagus

Otto 1841 provided the first brief description of a partially double-syncephalus pig [11]. During the following years, many cases of *syncephalus thoracopagus* were published. Glaser (1928) [11] published a detailed and extended account of swine cephalothoracopagus cases and discussed other similar cases described until then mainly by European scientists.

Table 2 presents brief descriptions of various malformations of *Syncephalus Thoracopagus* piglets. Also, in Figure 1c and Figure 2a–e some variations of *Syncephalus thoracopagus* are illustrated. Cephalothoracopagus is the most common case of conjoined twins in swine. 

### 2.4. Our Case

A case of two conjoined piglets born dead was submitted to the Faculty of Veterinary Medicine, School of Health Sciences, University of Thessaly by a local pig breeder from a farm of 450 sows. There had been no prior occurrences of conjoined twins on this farm and the sow had previously delivered multiple normal piglets. Labor comprising the conjoined twins began to proceed normally. The sow delivered live and healthy piglets at 5 to 10 min intervals. All live-born piglets had a birth weight of approximately 1.1 kg. The last cub followed, but they were conjoined twins. The labor became very complicated and lasted 12 h. The sow was then administered oxytocin at a concentration of 10 IU/mL to stimulate uterine contraction. Eventually, the dead conjoined twins were withdrawn with professional assistance. The female was subsequently excluded from breeding. The case of the conjoined twins was unique on this farm. 

**Figure 3:**
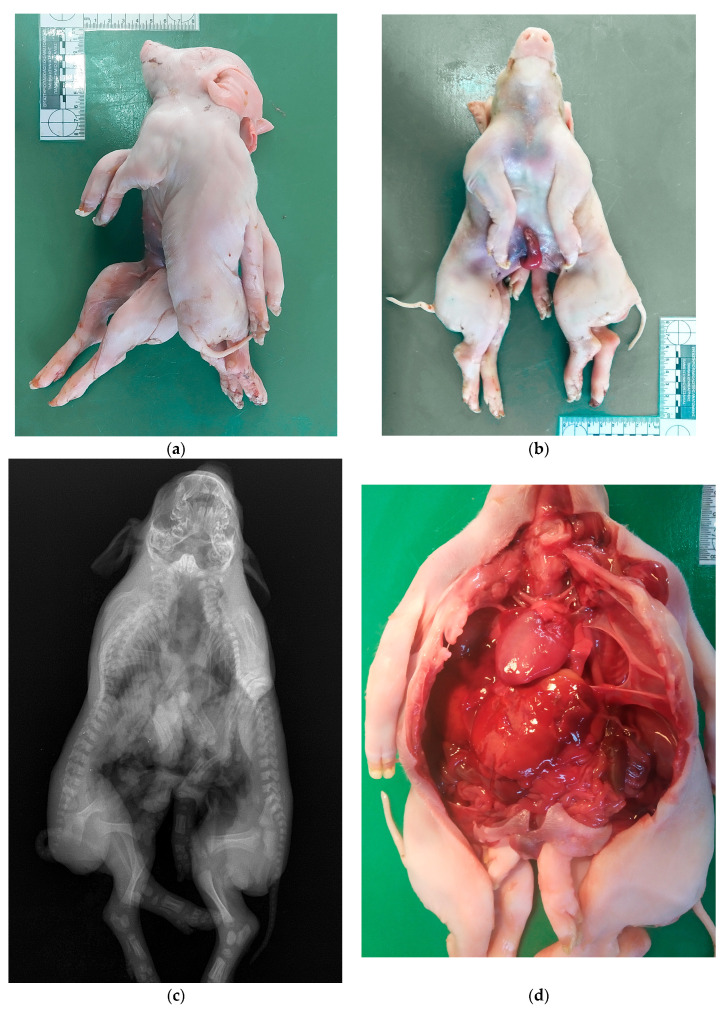
Studied case of the two conjoined piglets: the external gross view in a lateral (**a**) and in a supine position (**b**) before necropsy; a panoramic X-ray image in a supine position (**c**); and a panoramic gross view of the opened body cavities, showing the single enlarged heart and the other internal organs (**d**). The notable findings in each image are described in detail in the above text (Section 2.3.). Personal archive of Assistant Prof. Dimitrios Doukas.

The external gross examination revealed that the two piglets were joined at the head, neck, and thorax anatomic regions, forming one-headed conjoined twins with one oral cavity, two nostrils, two eyes, four ear pinnaes, one thoracic cavity, four forelimbs, four hindlimbs, two tails, and only one umbilicus (Figure 3a,b). The conjoined piglets were both female with a normal vulva and anus each.

Then, an X-ray examination of the dead conjoined twins was taken, showing one extended cranial cavity and one extended thorax with 13 ribs bilaterally, ending in one sternum. Moreover, two separated and complete spinal columns were detected (Figure 3c).

Finally, a full necropsy of the specimen was performed in the necropsy room. The presence of two completely separated bodies caudal to the single umbilicus was confirmed with normal kidneys and other retroperitoneal organs in each one piglet. In contrast, cranially to the single umbilicus, the below congenital conjunction patterns were found:

- Two separated spinal cords (one per each vertebral canal);
- A single oral cavity with a single esophagus ending up in a common stomach;
- A common abdominal cavity;
- A common duodenum dividing into a Y shape, creating two separated jejunum tubes;
- A single liver and a single pancreas;
- A single larynx with a single trachea ending up in two bronchi;
- Separated lungs (one lung per bronchus);
- A single enlarged heart (Figure 3d).

### 2.5. Thoraco-Omphalopagus

The conjoined twins which belong in this category are normally formed piglets joined by a union that involves the thoracic and abdominal region. The union extended from the umbilical cord up to the base of the neck [27] or the point of union was along the ventral surface of the body, extending from the first rib to a point just posterior to the umbilicus [28] or the fused abdominal cavities at the cranial third of the abdomen, and the thoracic cavities were completely fused at the sternum and had pairs of ribs [12]. The *thoraco-omphalopagi embryos* had a single umbilical cord for both piglets.

The double pig of Baumgardner and Everham (1936) [27] possessed separate and normal legs, tails, heads, and necks. All the main structures of the digestive tracts were normal, apart from the small intestines. These joined a short distance from the duodenum, after which there was a bifurcation, and the tracts were normal to their respective end. The most interesting feature of this piglet was the development of the bile ducts. Each duct entered the stomach instead of the duodenum. The livers were fused. 

Partial dissection of the swine specimen [28] revealed some interesting information concerning the arrangement of the internal organs and the region of fusion of the twins. The umbilical cord was composed of two complete sets of umbilical vessels. The sternums were completely absent, the animals were joined both at the ribs, all of which were fused, and along the anterior portion of the abdominal wall. This resulted in the formation of common thoracic and abdominal cavities, separated by a complete diaphragm. A single pericardium enclosed the two hearts, the apices of which overlapped. The livers formed a single organ; there was no definite line of demarcation between the two portions which had completely fused during development. One stomach was well-developed, while the other lacked a pyloric portion.

Postmortem examination of the two piglets [12] revealed them to be identical and symmetrical (Figure 1f). Completely separated heads and necks were observed in both piglets, with four ears, four eyes, four nostrils, and two mouths. Moreover, four duplicated and fully developed limbs were noticed. A separate abdominal and thoracic cavity was observed in each piglet, while the anatomical structures in one piglet were a mirror image of the other (similar and well-developed internal organs were noticed in both piglets). The livers were fused, with a separate gall bladder in each of them. A separate long, narrow spleen and stomach, two normal kidneys and ureters, and a urinary bladder were noticed in both piglets. In addition, a heart and a pair of lungs were observed in both piglets, with a common pericardial membrane. However, the heart and lungs were comparatively less well-developed in the one piglet [12].

## 3. Free Monozygotic and Parasitic Twins

A case of monozygotic asymmetrical twins, hemiacardius acephalus, was investigated [29]. The asymmetrical twins comprised a well-formed fetus and another which was defective. The *hemiacardius acephalus* lacked the head. The rest of the skeleton was formed and at the apical left part of the cervical region of the trunk, a rudimentary auricle with a blind ear canal was detected. An *epigastricus* parasite twin is illustrated in Figure 1e.

## 4. Discussion

Based on the literature, numerous abnormal twin classifications have been reported, based on anatomy, site of union, symmetry level of twins, and embryological development. Abnormalities in the anatomy of conjoined twins arise during the prenatal period of the pregnant animals. The implications of the aforementioned abnormalities can lead to organ dysfunction or failure and often death. However, in regard to the main causes of the development of conjoined twins, several genetic and environmental factors are proposed [4].

The origin of conjoined twins has been described by two main theories [30]. The first theory assumes that a fertilized egg splits into two embryos in the early stages of cell division. However, the individual embryos do not split completely, as is the case in the normal development of identical twins, but some body parts of the two siblings remain joined. In the development of conjoined twins, the embryo only partially separates to form two bodies, but conjoined twins are a single organism with multiple morphological duplications. Although the two fetuses develop, they remain physically joined, usually at the breast, abdomen, or pelvis. The second theory is that the two embryos are initially completely separated, but then the active stem cells find each other and join to form a new adhesion of different sizes and locations [31].

In the cases surveyed in the present review, most conjoined piglets were male and in only two of them [12,18] they were female. The imperfect embryonic twinning of piglets has occurred independently of the breed. The cumulative observations of the current survey are from the 67 records by Selby et al. (1973) [10]. The frequency of conjoined twinning is reported as 6/1000, or 0.6%, and it is considered the most common defect (without consideration of multiple anomalies), representing 51/319, or 8%, of the total number of abnormal newborn piglets [32]. In the same survey, among 57 piglets with cleft palates, 21 (36.8%) were classified under conjoined twins. In another survey of swine congenital malformations, among one hundred and seventy-four defects, four were reported as conjoined twins [32]. However, a previous study supported that the recalculation from these conservative estimates indicates that the conjoined twinning rate is approximately 0.048% of all swine births [9]. The diprosopi-dipygus animal is an extremely rare abnormality for swine (1.5% of swine-conjoined twins or 0.00072% of swine births) [10]. The frequency of conjoined twinning in different geographic areas is not fully understood. For example, no conjoined twins were noticed in a study carried out in Denmark including 29,886 births [33].

The report of each case of abnormal twins from veterinarians, farmers, and swine breeders is a crucial point. For future studies, it is very important to have access to appropriate data from farmers or swine breeders about the grandparent sows, genetic background, animal welfare conditions, nutrition, vaccinations, health status, infectious diseases, etc.

## 5. Conclusions

To date, several cases of congenital malformations in swine have been reported. The occurrence of newborn conjoined twins is always very interesting and unique. These anomalies are often observed during or after perinatal reproductive disorders (e.g., dystocia), mainly due to poor prenatal screening techniques in swine. Therefore, congenital malformations are an important pathological condition in pigs, and each case should be studied as an individual case because it presents particular anatomical differences. The issue of this anomaly has not yet been clearly explained. Due to the large number of newborns, it seems likely that genetic factors are negligible in the occurrence of congenital anomalies. However, breeding conditions can also be hypothetically included among the causes. For future studies, it is necessary to have access to appropriate data from farmers or pig breeders to be able to identify the pathogenetic mechanisms leading to this condition and, thus, prevent the occurrence of these anomalies.

## Figures and Tables

**Table 1:** The classification of abnormal twins according to the *Nomina Embryologica Veterinaria*.

Geminus acardiacus			
	Defectio cordis		
		totalis	
		subtotalis	
Gemini conjuncti			
	Gemini symmetrici		
		Junctio cranialis	
			dorsalis
			lateralis
			ventralis
		Junctio media	
			xiphoidea
			sternalis
			thoracica
			thoraco-epigastrica
		Junctio caudalis	
			dorsalis
			lateralis
			ventralis
	Gemini asymmetrici		
		Junctio cranialis	cranialis parasitica
			gnathialis parasitica
		Junctio media	
			thoraco-epigastrica parasitica
			abdominalis parasitica
		Junctio caudalis	
			pygalis parasitica

**Table 2:** Brief descriptions of various malformations of *Syncephalus thoracopagus*.

Classification	Brief Description	References
*Syncephalus thoracopagus*	Partly fused bodies. A single head. Two lateral ears and a median one. Three nostrils on the snout. Doubling in the brain: a third hemisphere between the right hemisphere of one brain and the left hemisphere of the other brain. A distinct cerebellum. A spinal cord to each of the lateral hemispheres. Two sets of forelimbs and hindlimbs. Unsymmetrical organs on the median line may be taking place in any double organs (e.g., eyes, ears, legs, arms, lungs, kidneys, etc.).	Wyman 1861 [14]
	Two bodies sideways with respect to the bodies. A single head. Double tongue and lower jaw. Eight legs.	Wilder 1908 [5]
	A normal head (more nearly with two ears). Two eyes. Two nostrils. An alimentary canal. A divided pharynx. A grooved stomach. A single pancreas and spleen. A single trachea: connection to a ventrally lobed lung mass. A fused heart: single venous system. Arterial supply of the head: one side of the heart only. A fused nervous system: complete separation in the spinal cords and fusion from the medullae anteriorly.	Williams and Rauch 1917 [15]
*Syncephalus thoracopagus*	Two fused heads with two eyes. One snout with four nostrils. The fusion of the bodies extended to the umbilicus. A single umbilical cord with four arteries and two veins. A proboscis of a cyclops on the dorsal aspect of the fused heads. One large heart situated ventrally within the thorax in a separate pericardium. A separate pericardium in the smaller one. A ventral and dorsal pair of lungs: each pair with a corresponding trachea leading to a larynx. A compound tongue. A single alimentary tract within the cecum. Bifurcated ileum at the ileo-cecal valve. Two livers: the right being larger than the left. Double genito-urinary system: normal. Undescended testicles. Two spleens. Two encephali: distinct brain, stem, and spinal cord.	Carey 1917 [16]
	Four larynges: an epiglottis in each. Three lower jaws: a tongue and a full set of teeth in each. One quadruplex larynx. One proximally bifurcated esophagus, communicating with a large common pharyngo-oral cavity. Two maxillae opposed to the three mandibles. Two distinct cerebrospinal systems. Two distinct skeletons, except for fused crania and sterna. Two tracheae and pairs of lungs (equal size placed back-to-back). Two hearts (very unequal size) with dorsal surfaces opposed. Two aortae and two inferior venae cavae: communication with the larger heart. A common liver and one common spleen. A compound stomach, one small intestine, and two colons. One compound thymus. Two pancreata. Two complete male genito-urinary systems. Three eyes, including a single median eye: two optic nerves entering at a common disc. Two ears. A common umbilical cord. Eight legs: two placed mediodorsally.	Jordan et al. 1923 [17]
	One head. A normal mouth, nares, and eyes. Four external ears: two on the mid-dorsal line just back of an unexplored wart-like protuberance in the pineal position. Eight normal legs. Joined bodies (one much larger): by ventral surfaces in the thoracic regions. A common metacoele. Most viscera in the body of the larger twin, or in the common portion. A complete digestive tract (larger twin). An incomplete digestive tract (smaller twin). A relatively short intestine: into a pouch in the intestine (larger twin). Two pleural cavities. Two sets of lungs dorsal side to dorsal side. Two tracheae: the ventral one uniting with the single esophagus near the larynx. Two hearts: dorsal surfaces opposed. Two dorsal aortae: connection with each other and with the hearts. Four jugulars: two precavae and two postcavae, with an unconnected venous system. Normal kidneys and ovaries with normal ducts and blood connections (the larger twin). Rudimentary sex organs and kidneys (smaller twin). Dorsal vertebral columns: connection with the thoracic regions. Great glandular development, especially of the endocrine glands. Greatly developed thymus.	Hunter and Higgins 1923 [18]
*Cephalothoracopagus monosymmetros*	A ventrolateral union. The snout and mouth were broad and single. Two eyes, two ears, and four fore legs. A single umbilical cord with four arteries and two veins. Two livers: one large and one small. A single esophagus. A fused double stomach. The intestine bifurcated. Two glottidae. Two tracheae. Two hearts: each in its own pericardium. Both aortae from one heart. A complete absence of at least the lower ends of both posterior venae cavae. A complete lack of fusion of the two brains. An absence of any connection between the two cranial cavities.	Baumgardner 1928 [19]
*Syncephalus thoracopagus*		Nordby and Taylor 1928 [20]
*Cephalothoracopagus monosymmetros tetrophthalmus synotos tetrabrachius*	Conjoined female twins. A head with two faces: a normal and a defective with synotia and incomplete cyclopia. Each face with a mouth cavity. Two thoracic cages with 15 pairs of ribs each. Double throat organs, except the esophagus. Hypoplastic lungs of the one twin. Two hearts, spleens, pancreata, and livers. One stomach in the common abdominal cavity. A single duodenum and jejunum which divided in double ileum, colon, and rectum. Double normal urogenital tract.	Glaser 1928 [11]
	Conjoined twins, completely haired, and male. Eight teeth. An umbilical cord with four arteries, one vein, and two allantoic ducts. Two livers: the second much smaller than the first. Unpaired stomach the size of a chicken egg. Small intestine divided in two colons filled with meconium.	Glaser 1928 [11]
*Cephalothoracopagus monosymmetros synotos tetrabrachius*	Two uniform, well-haired male individual parts. An umbilical cord with all vessels twice. A completely immersed stomach in the centrum tendineum of the diaphragm merged with it.	Glaser 1928 [11]
	Two equal-sized male individual densely hairy parts. An umbilical cord with four arteries, one vein, and two allantoic ducts.	Glaser 1928 [11]
	Very uniformly formed, well-haired, and male. An umbilical cord with seven vessels. Simple umbilical vein.	Glaser 1928 [11]
	Male, slightly hairy. Double number of umbilical vessels. Eight teeth in a formed face. Angle of rotation: 68 degrees.	Glaser 1928 [11]
*Cephalothoracopagus monosymmetros biauritus tetrabrachius*	Comprised twins of two individual male parts: overweighted left part. An umbilical cord with all vessels twice.	Glaser 1928 [11]
	Symmetrical double formation: two male, well-haired individual parts. An umbilical cord with seven canals, four arteries, a vein, and two allantois ducts. A regular face, except for a cleft palate and eight milk teeth. Closed spines at the neck to each other. Separated spines from the first thoracic vertebra under strong kyphotic bending.	Glaser 1928 [11]
	An umbilical cord with four arteries, a vein, and double allantois ducts. A head with two foramina magna.	Glaser 1928 [11]
*Cephalothoracopagus monosymmetros synotos tetrabrachius cranii e latere coalitis*	Lateral adhesiveness of the skull. An umbilical cord with all vessels. Two allantois ducts, inserting at the most caudal part of the adhesion. Two hearts. Completed descent of the testicles.	Glaser 1928 [11]
*Prosopothoracopagus disymmetros*	Twins joined ventrally: from the navel to the oral cavity. Common oral cavity. Normal genito-urinary system organs: each part.	Glaser 1928 [11]
*Cephalothoracopagus monosymmetros*	One head. Eight legs. Duplicated trunk at the level of C3 vertebra.	Engel 1931 [21]
	One head with two eyes. One snout and one tongue. Two tracheas originated from two larynxes. A centrally located, single esophagus continued into a stomach. Same urinary and gastrointestinal tract in both twins. Two separated spinal columns in their entire course towards two foramina magna.	Engel 1931 [21]
*Cephalothoracopagus*	One twin smaller than the other. Well-developed twins. A single head with one snout and two eyes. Four ears: two fused ears at the external acoustic meatus. A proboscis-like structure (anterior to the fused ears—approximately 1.5 cm). Two forelimbs and hindlimbs (per each twin) in apparently normal position. An anal opening and a penis (per each twin). Two pairs of parotid glands. Two livers: both of which were attached to the common diaphragm and common stomach. A single esophagus into the stomach, as resulting from the fusion of two stomachs at the median line. A single small intestine and two large intestines, because of branching anterior to the entry of the ileum into the cecum. Double genito-urinary organs. Two hearts: a large and a small in separate pericardia.	Hetzer and Eaton 1943 [22]
	A single head and two partially fused bodies. Vertebral columns arose from the double occiput. Completely separated bodies caudal to the single umbilicus. Four normal limbs. Normal male external genitalia. A single buccal cavity. A protruded tongue through a cleft palate into the ventral nasal meatus. A single esophagus. Double larynx and tracheae. Four lungs. Double digestive tract in the small intestine, transforming Y fashion into two tubes. A common sinus venosus, communicating with both sides of the single heart (in the middle of the four pleural cavities). A pulmonary artery with ductus arteriosus terminating as the aorta of the left twin. An aorta giving rise to two common carotids without a brachiocephalic trunk. Four umbilical arteries, two from each side, entered the cord. Two spinal cords, medullae, cerebelli, and thalami.	Halnan 1970 [23]
	Complete rostrally ventral fusion, extending to a point just caudal to the single umbilical cord. Two complete sets of normal limbs. Four ears: two situated laterally and two conjoined dorsally (normal, no ear canals). Three eyes: two laterally in the normal position and one (highly underdeveloped) just rostral to the conjoined ears. Duplication of most of the viscera. A peculiar arrangement of the hearts. Esophagus and stomach as singular structures. Normal cerebral hemispheres. Duplicated cerebelli, spinal cords, and cranial nerves VIII-XII. Duplication of the skeleton caudally from the middle cranial fossa.	Czarnecki and Schenk 1977 [24]
*Cephalothoracopagus monocephalic dithoracic*	A single head with neck and chest. Four forelimbs and four hindlimbs. Two complete vertebral columns. Two tongues and a cleft palate. Two larynxes and tracheas and a single pharynx. Single esophagus, stomach, duodenum, and part of jejunum. Single spleen, liver, and pancreas. A separate urogenital apparatus. A heart with structural abnormalities. Two lungs: right lung in the right twin/left lung in the left twin.	Kulawik et al. 2017 [25]
*Prosopothoracopagus*	One smaller twin than the other. Well-developed twins. Fused bodies to embrace the thoracic and cervical regions and the head. United xiphoid processes by cartilage.	Lloyd Jones 1945 [26]

## Data Availability

Data is unavailable due to privacy.
